# Evidence That HIV-1 CRF01_AE Is Associated with Low CD4+T Cell Count and CXCR4 Co-Receptor Usage in Recently Infected Young Men Who Have Sex with Men (MSM) in Shanghai, China

**DOI:** 10.1371/journal.pone.0089462

**Published:** 2014-02-21

**Authors:** Xiaoshan Li, Yile Xue, Leiming Zhou, Yi Lin, Xiaolei Yu, Xuqin Wang, Xiaohong Zhen, Wei Zhang, Zhen Ning, Qing Yue, Jie Fu, Fangwei Shen, Jing Gai, Yuqing Xu, Jiawen Mao, Xianming Gao, Xiaopei Shen, Laiyi Kang, Guido Vanham, Hua Cheng, Ying Wang, Minghua Zhuang, Xun Zhuang, Qichao Pan, Ping Zhong

**Affiliations:** 1 Department of AIDS and STD, Shanghai Municipal Center for Disease Control and Prevention, Shanghai, China; Shanghai Municipal Institutes for Preventive Medicine, Shanghai, China; 2 Public Health College, Nantong University, Nantong, China; 3 Immunovirology Group, Biomedical Science Department, Institute of Tropical Medicine, Antwerp, Belgium; Fudan University, China

## Abstract

Men who have sex with men (MSM) have recently accounted for an alarmingly increasing proportion of HIV-1 transmission in China. In order to investigate the immune status as a result of CRF01_AE infection and CXCR4 co-receptor usage in a young Shanghai-based HIV-1-infected MSM population in Shanghai, 364 HIV-1-infected MSM with average age of 22.7 years old, newly diagnosed between Jan 2009 and Jul 2013 were analyzed for CD4+T cell count, subtyping using phylogenetic analysis, and viral co-receptor tropism using Geno2pheno and webPSSM in combination. A total of 276 individuals were identified as recently infected. Subtype assignment were as follows: 176 (63.8%) CRF01_AE, 77 (27.9%) CRF07_BC, and 23 (8.3%) subtype B. Besides, 24 second-generation recombinant strains were identified. A lower CD4+T cell count at baseline survey was observed among CRF01_AE strain-infected individuals, compared to those who were infected with CRF07_BC (*P*<0.01). The frequency of baseline CD4+T cell count <200 was higher and the frequency of CD4 T counts >500 lower in CRF01_AE infection than CRF07_BC infection. It is worth noting that 32.4%–40.9% of CRF01_AE strain-infected individuals were predicted to carry CXCR4-tropic viruses whereas none of CRF07_BC and subtype B were found to be as CXCR4-tropic viruses (*P*<0.001). As could be expected CXCR4 tropism was associated with lower CD4 T counts. This study revealed that CRF01_AE strains with high frequency of CXCR4 tropism are prevailing in the young MSM population in China and could potentially cause a severe loss of CD4+T cell count and rapid disease progression. A regular surveillance of HIV-1 subtypes, CD4+T cell count and viral co-receptor usage would be greatly beneficial for effectively monitoring disease progression, improvement of antiretroviral therapy strategy and prompt intervention of transmission.

## Introduction

Men who have sex with men (MSM) have now become the most vulnerable risk group to HIV-1 infection in China. By the end of 2011, the estimated number of people living with HIV (PLHIV) in China stood at 780,000 [1]. Among the 58,399 newly diagnosed HIV-1 infections in 2012, 64.3% could be attributed to heterosexual and 21.6% to homosexual transmission (from a report of National Center for AIDS/STD Control and Prevention, China CDC). Compared with the estimated 2.5% in 2006, 13.7% in 2011, homosexual transmission has thus become a very significant mode of transmission for new HIV infections in China [1].

A meta-analysis of twelve relevant studies revealed that the sub-overall HIV-1 incidence estimates were 3.5% (95% CI: 1.7%–5.3%) and 6.7% (95% CI: 4.8%–8.6%) among MSM in China for cohort and cross-sectional studies, respectively [2]. The sentinel surveillance found that overall HIV prevalence among MSM over the years showed a rising trend from 0.9% in 2003 to 6.3% in 2011 [3]. A cross-sectional survey among 47,231 MSM from 61 cities in China conducted from February 2008 to September 2009 indicated that the overall prevalence of HIV infection was 4.9% [4].

Nationwide molecular epidemiologic surveys and other studies have revealed that CRF01_AE strain has been overtaking the subtype B in MSM population in recent years [5], [6], whereas it was initially prevailing in heterosexual population in eastern coastal areas and southwest border provinces [7], [8]. Based on the recently unpublished information, a high proportion of reported AIDS cases were discovered in the newly diagnosed infections during recent years (22.3% in 2007, 19.1% in 2008, 21.8% in 2009, 25.0% in 2010, 27.9% in 2011 and 29.2% in 2012). This has usually been attributed to a delayed diagnosis. However, another possible explanation is that highly pathogenic viruses may circulate and cause rapid disease progression. The effect of HIV-1 subtypes or recombinants on clinical outcome has been reported [9], [10]. Several studies also have demonstrated a high prevalence (31.3%–61.5%) of CXCR4-tropic virus among treatment-naive or newly diagnosed CRF01_AE-infected individuals [11], [12]. However, there are very few investigations on the relationship between genetic subtypes, immune status, and co-receptor tropism among HIV-1-infected MSM individuals in China.

As a Chinese coastal international metropolis, Shanghai attracts increasing numbers of migrant populations every year because of the booming economy, cultural diversity and the open mind in this city. The continuous migration waves have brought a high prevalence of HIV-1-infection in Shanghai [3], and HIV-1 infection among floating population dominated all new HIV-infections every year (such as 70.7% in 2009, 62.1% in 2010, 68% in 2011 and 70.7% in 2012, from a report of Shanghai CDC). Since the first HIV-1 case was found in 1987 in Shanghai, the cumulative number of reported HIV-1-infected persons has reached 9324 in 2012. From the estimated 18 million men engage in homosexual sex in China, approximately 80,000 MSM were living in Shanghai and the HIV prevalence among MSM showed a rising trend from 1.5% in 2004 [13] to 6.8% in 2009 [4] and 7.5%–8.0% in 2011–2012 (from a report of Shanghai CDC). We therefore carried out a preliminary study in Shanghai aiming to elucidate the impact of viral genetic diversity and co-receptor usage on disease progression in young MSM who had recent HIV-1 infection during the period between January 2009 and July 2013.

## Materials and Methods

### Study Subjects

Three-hundred and sixty-four HIV-1-infected MSM individuals between age 18 and 25 living in Shanghai and newly diagnosed between January 2009 and July 2013, were analyzed retrospectively.

HIV-1 infection was screened by HIV-1/HIV-2 enzyme-linked immunosorbent assay (Vironostika, Organon Teknika, Oss, The Netherland) and confirmed by Western blot HIV-1 (HIV blot 2.2, MP Diagnostics, Singapore). Plasma was recovered from EDTA anti-coagulated blood, collected for CD4+T cell counting within about 3–6 months after infection had been confirmed.

Acute infection was defined as the period between exposure to the virus and completion of the initial immune responses, i.e. by detectable HIV RNA in plasma in the setting of a negative or indeterminate HIV antibody test. Primary and recent infections were defined as the period between 6 and 24 months following the exposure to the virus, respectively. Chronic infection was defined as evolving for more than 24 months after the viral exposure [14]. In order to identify recent infection amongst these 364 MSM, we combined an epidemiological and a molecular approach. First, to “enrich” the studied MSM population for recent infection we focused on a young age group (under age 25, mean age: 22.7) [15], since the first time sex exposure among Shanghai’ MSM has been shown to occur between 20–21y [16], [17], and the proportion of the first time sex exposure among MSM ≤25 years old was 78.1% [18]. Second, a molecular algorithm was applied to narrow down on recent infections: it has been shown that a frequency of ambiguous calls in bulk sequencing of *pol* gene under 0.44% might distinguish recent infection from long-standing infection [19], [20].

The study was reviewed and approved by the Institutional Review Board at the Human Medical Research Ethics Committee of the Shanghai Municipal Center for Disease Control and Prevention. The Board decided to waive the need for written informed consent from the participants studied in this project based on the characteristics of this study project. No informed consent from participants was obtained as the data were analyzed retrospectively and anonymously.

### CD4+ Lymphocyte Counts

Patients’ blood samples were collected using an EDTA Vacutainer (Becton and Dickinson Company, USA). CD4+ T lymphocyte counts were measured in our laboratory by flow cytometry (FACS Calibur, BD Company, USA) within 24 h. The fluorochrome conjugated antibodies for four-color cytometry were anti-CD3, CD4, CD8, and CD45 (Becton Dickinson, San Jose, California and Pharmigen, San Diego, California, USA). The daily quality control for CD4+T cell counting was performed using LymphoSure (Synexa, Life Science, South Africa). The blood samples were then centrifuged at 2500 rpm for 10 min to separate plasma and buffy coat. Plasma was frozen in multiple aliquots at −80°C until use.

### RNA Extraction and RT-PCR Amplification

HIV-1 genome RNA was extracted from 200 µl of stored plasma specimens using the QIAmp Viral RNA Mini kit (Qiagen, Valencia, CA, USA) as Manufacturer’s instructions. Reverse transcription and nested polymerase chain (nPCR) amplification for partial genes of *pol* and *env* were performed by a home brew PCR procedure as described in our previous reports [6], [21]. A one-tube reverse transcriptase polymerase chain reaction kit (GoldScript one-step RT-PCR kit, Life Technologies, USA), and PCR kit (TaKaRa Ex Taq Kit, Takara Biotechnology Co, Ltd; Dalian, China) were used according to the manufacture’s recommendations for amplification of the HIV-1 *pol* gene (protease 1–99 amino acids and part of reverse transcriptase 1–254 amino acids) and *env* gene (part of gp120 C2V5, 220 amino acids). About 1050 bp *pol* and 660 bp fragments were amplified. The PCR amplification was carried out in a thermal cycler (GeneAmp PCR System 9700, Applied Biosystems, USA). PCR products were directly sequenced in both directions with sequencing primers using ABI 3730 sequencer. Pre-PCR and post-PCR areas are strictly separated in order to avoid contamination from amplicon aerosol.

### Phylogenetic Analysis based on *env* and *pol* Genes

The resulting gene fragment sequences were aligned with reference sequences of various subtypes from the Los Alamos HIV-1 database. Multiple alignments were made automatically using the Bio-Edit version 5.0 with minor manual adjustments. A phylogenetic tree was constructed by the neighbor-joining method implemented by MEGA version 5.0. The Kimura two-parameter method was used for the determination of the evolutionary distance. The reliability of the branching pattern was assessed by bootstrap analysis with 1000 replicates. All the nucleotide sequences obtained were screened by the HIV-BLAST (http://www.hiv.lanl.gov) to search for sequences in the databases and rule out the potential laboratory errors.

### Prediction of Viral Co-Receptor Usage

Viral sequences were analyzed for co-receptor usage based on V3 loop sequences, using two online tools: webPSSM: http://fortinbras.us/cgi-bin/fssm/fssm.pl, and Geno2Pheno: http://coreceptor.bioinf.mpi-inf.mpg.de/. These two analysis tools are all available for using the nucleotides sequences containing more sequence peaks at the same location. European Guidelines for HIV patient management currently recommend the use of Geno2Pheno with a 10% false positive rate (FPR) cut-off, which has been shown to provide the best balance between specificity and sensitivity for predicting CCR5 or CXCR4 tropism [22]. As both Geno2Pheno (FPR = 10) and PSSM were thought to overestimate the presence of CXCR4 viruses for CRF01_AE [23], we adopted the recently published algorithm that uses both Geno2pheno (FPR = 10%) and PSSM in combination (algorithm I), with 88.9% of sensitivity and 89.3% of specificity [24]. Tropism measured was considered to be concordant only if both algorithms detected pure CCR5 or pure CXCR4 co-receptor usage. Besides, we also simultaneously using an algorithm of Geno2pheno (FPR = 5%) and PSSM in combination (algorithm II) in order to improve the analytic specificity and obtain more precise conclusion.

### Statistical Analysis

Two Independent Samples Nonparametric Tests (Man-Whitney U) was used for statistical analysis of the relationship between CD4+T cell count and genetic subtypes and viral tropisms. Fisher’s exact test was used to calculate the tropism genotypic distributions among various subtypes, years, and CD4+T cell count groups. *P* values less than 0.05 were considered as statistical significance. All statistical analyses were performed using SPSS v.16 software (IBM Company, New York, USA).

## Results

### HIV-1 CRF01_AE Strains Dominate HIV-1 Epidemic in Young MSM of Shanghai

Three hundred and sixty four MSM between 18 and 25 (average: 22.7) were found to be newly HIV-1 infected between 2009 and 2013. None of them were treated with antivirals at the time of investigation. Based on a low frequency of ambiguous calls in *pol*, 276 of those were considered as recent infections. All these subjects belong to Han ethnicity, 19.9% (55/276) are Shanghai native, 78.6% (217/276) belongs to migrant population, and the others 1.5% (4/276) are unknown (Table 1).

**Table 1:** Socio-demographic Characteristics of Studied Subjects Based on Subtypes.

Patients	CRF01_AE (%)	CRF07_BC (%)	Subtype B (n)	*P* value
Years*				
2009 (n = 58)	36(62.1)	12(20.7)	10(17.2)	0.615
2010 (n = 57)	41(71.9)	11(19.3)	5(8.8)	
2011 (n = 59)	37(62.7)	18(30.5)	4(6.8)	
2012 (n = 75)	49(65.3)	22(29.3)	4(5.3)	
2013 (n = 27)	13(48.1)	14(51.9)	0	
Ethnicity (Han)	176(100.0)	77(100.0)	23(100.0)	–
Age (average)	22.8±1.9	22.6±2.1	22.3±1.9	0.480
CD4+ T cell counts (cells/µl)	415.5 (274.3–537.0)	476.5 (339.3–617.5)	475.0 (351.0–590.0)	0.013
Regions**				
Shanghai (n = 55)	32(58.25)	18(32.7)	5(9.1)	–
North (n = 9)	4(44.4)	2(22.2)	3(33.3)	
Northeast (n = 8)	6(75.0)	2(25.0)	0	
East (n = 118)	83(70.3%)	30(25.4)	5(4.2)	
South Central (n = 34)	22(64.7)	6(17.6)	6(17.6)	
Southwest (n = 39)	26(66.7)	10(25.6)	3(7.7)	
Northwest (n = 4)	2(50.0)	1(25.0)	1(25.0)	
Unknown (n = 9)	1(11.1)	8(88.9)	0	
Total (n = 276)	176(63.8)	77(27.9)	23(8.3)	

*The year when blood samples were collected.

**Place of birth. North: Beijing, Hebei, Shanxi and Inner Mongolia; Northeast: Liaoning, Jilin, and Heilongjiang; East: Fujian, Shandong, Zhejiang, Anhui, JiangXi, and Jiangsu; South Central: Guangxi, Henan, Hubei, Hunan, Guangdong and Hainan; Southwest: Sichuan, Chongqing, Guizhou and Ynunan; Northwest: Shannxi, Gansu, Ningxia, Qinghai and Xinjiang.

**Figure 1:**
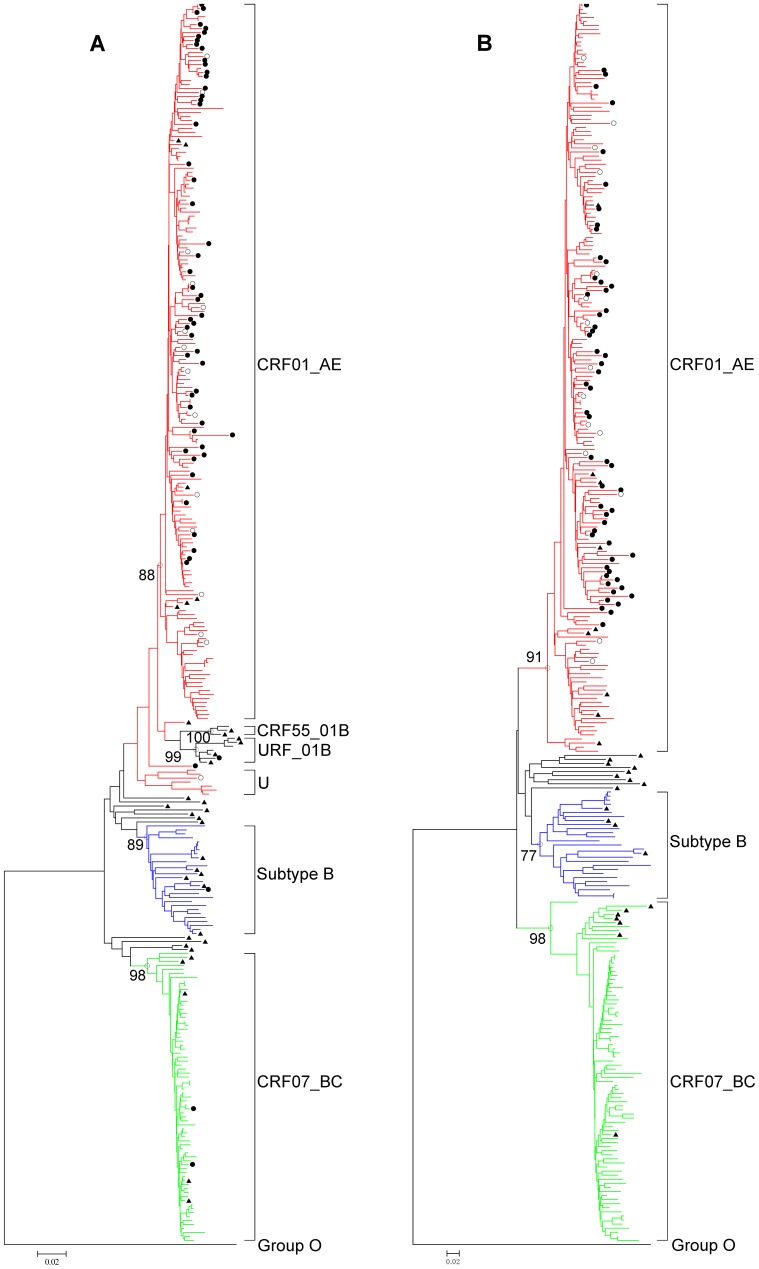
Phylogenetic tree analysis of HIV-1 *env* and *pol* gene sequences among MSM with recent infections in Shanghai. The phylogenetic trees were constructed using neighbor-joining methods (Mega 5.0) based on *pol* (A) and *env* (B) sequence regions. The bootstrap values of 1000 replicates above 75% are labeled on the major clusters nodes. The CXCR4-tropic strains determined with algorithm I were indicated by both solid and open circles, whereas the CXCR4-tropic strains determined with algorithm II were only indicated by solid circles. CRF01_AE sequences are marked in red, CFR07_BC sequences are marked in green, and subtype B/B’ sequences are marked in blue. U stands for unidentified subtypes/recombinants. The subtype reference sequences from the Los Alamos HIV sequence database (http://hiv-web.lanl.gov/content/index) were indicated by solid triangles. Trees were rooted using group O as a out group.

Phylogenetic tree analysis of HIV-1 circulating in 18–25 years old MSM in Shanghai indicated that belongs CRF01_AE strains were most frequent (63.8%, 176/276), followed by CRF07_BC (27.9%, 77/276) and subtype B or B' strains (8.3%, 23/276) (Fig. 1.A and B). In addition, 8.69% (24/276) discordant subtypes were identified, including 07_BC^pol^01_AE^env^ (n = 3), URF01B^pol^01_AE^env^ (n = 3), B^pol^01_AE^env^ (n = 1), 55_01_B^pol^01_AE^env^ (n = 1), 01_AE^pol^07_BC^env^ (n = 7), 01_AE^pol^B^env^ (n = 2), U^pol^BC^env^ (n = 5), and U^pol^01_AE^env^ (n = 2). In addition, in the tree based on *pol* gene, 3 (16.7%) sequences were found to be clustered with a unique 01_B recombinant strain and 1 (5.6%) was clustered with CRF55_01B, a recently identified strain in China (Fig. 1.A). One unidentified cluster with 7 sequences, found in the *pol* tree, was clearly close to the CRF01_AE cluster, but identified as CRF07_BC by HIV BLAST-based subtyping, although it remains however apart from CRF07_BC cluster in the tree. These unique clustering sequences would be a new recombinant and will be further analyzed.

### CRF01_AE Infection Leads to Low Level of CD4+T Cell Count

CD4+T cell count is still the strongest independent predictor of disease progression. Overall, in the 276 recently infected persons, only one-third (35.1%, 97/276) had a CD4+T cell count ≥500 (620 [IQR: 542–812]) cells/µl, whereas nearly 10% (9.5%, 26/276) had CD4+T cell count ≤200 (126.5 [IQR: 79.8–161.8]) cells/µl. Approximately 54% persons had CD4+T cell count between 200 and 499 cells/µl, of whom 24.6% (68/276) had 200 to 349 cells/µl, and 29.7% (82/276) had 350 to 499 cells/µl.

No significant difference was found for stratified CD4+T cell count based on median age (≤200∶22.54±1.5; 200–349∶23.2±1.8; 350–499∶22.8±2.0; and ≥500∶22.3±2.1. F = 2.982, *P* = 0.320), and sampling years as well (χ^2^ = 13.229, *P* = 0.353).

**Figure 2:**
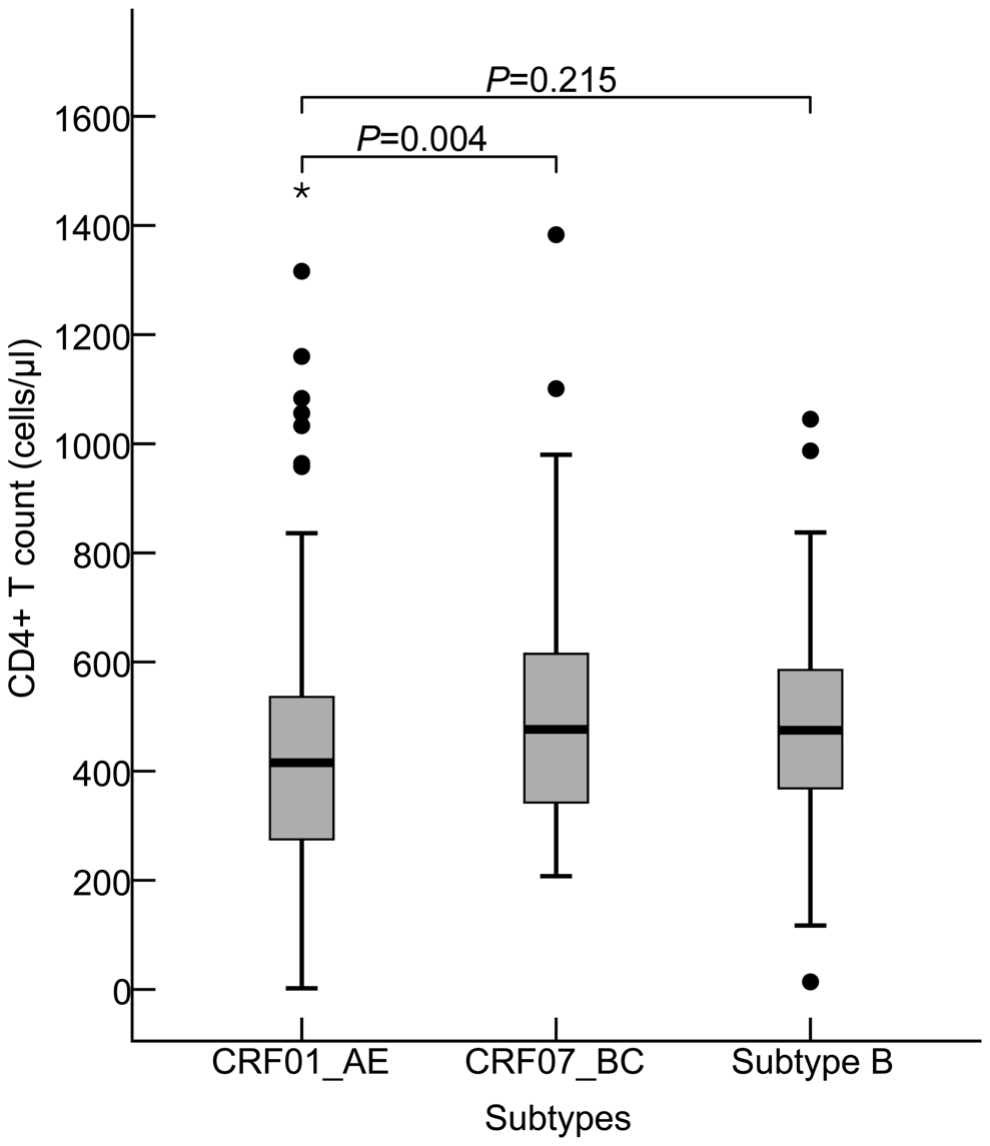
The levels of CD4+T cell count in Shanghai young MSM infected with HIV-1 CRF01_AE, CRF07_BC, and subtype B. The statistical significance in levels of CD4+T cell count (Median and Interquartile Range [IQR]) among three different subtypes and recombinants, CRF01_AE, CRF07_BC, and subtype B was calculated using the Two Independent Samples Nonparametric Tests (Man-Whitney U).

**Figure 3:**
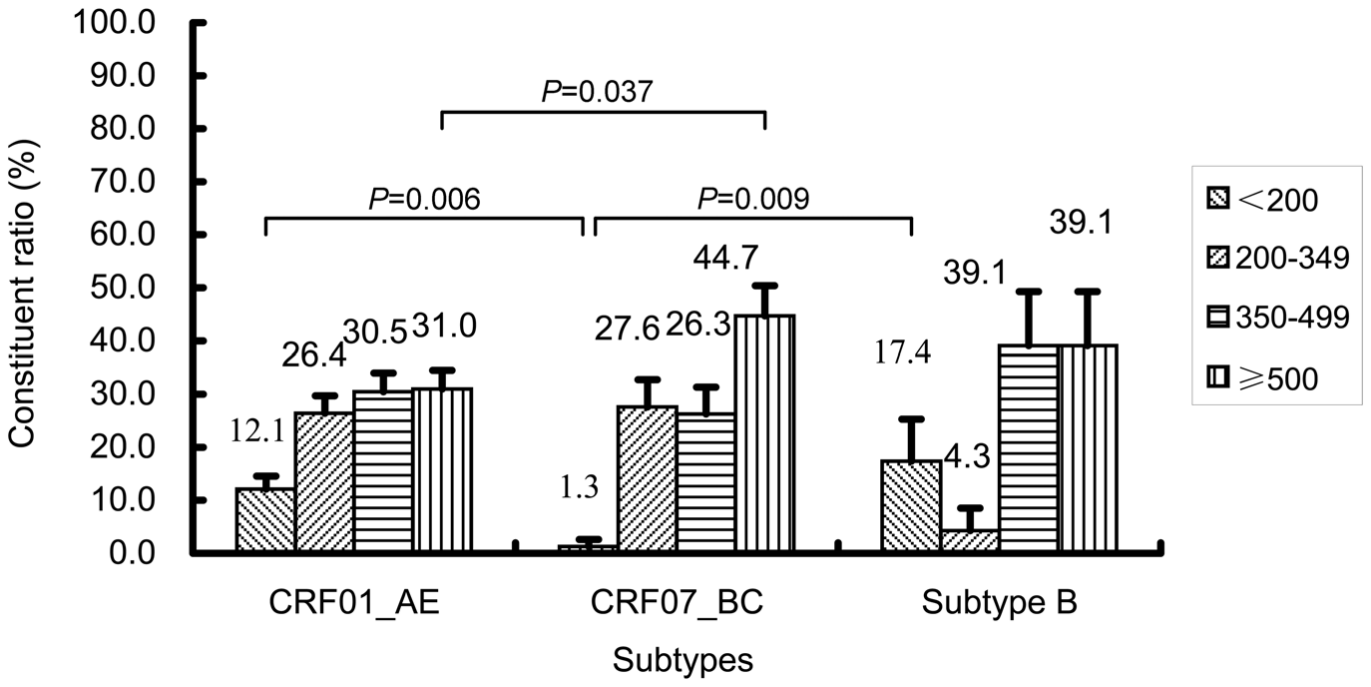
A strong association observed between HIV-1 CRF01_AE- and CRF07_BC-infected MSM, based on the analysis of stratified baseline CD4+T cell count. The statistical significance of the association between HIV-1 CRF01_AE and CRF07_BC infections based on the stratified baseline CD4+T cell count was evaluated using the Fisher’s exact test. The data are shown as the constituent ratio (95%CI).

An unexpectedly low level of baseline CD4+T cell count was found among 176 CRF01_AE-infected persons, compared to those who infected with 77 CRF07_BC (Z = 2.878, *P* = 0.004), but not to 23 subtype B (Z = 1.239, *P* = 0.215). The median CD4+T cell count in three subtypes-related groups were 415.5 (IQR: 274.3–537.0) cells/µl, 476.5 (IQR: 339.3–617.5) cells/µl, and 475.0 (IQR: 351.0–590.0) cells/µl, respectively (Fig. 2). A strong association was observed between two subtypes, CRF01_AE and CRF07_BC, based on the stratified baseline CD4+T cell count, (Fig. 3). The proportion of CRF01_AE infected subjects with CD4+T cell count ≤200 cells/µl was remarkable higher than in CRF07_BC infection (χ^2^ = 7.621, *P* = 0.006) and conversely the proportion of CRF01_AE with CD4+T cell count ≥500 cells/µl (χ^2^ = 4.354, *P* = 0.037) was lower than in CRF07_BC infected subjects (Fig. 3). This preliminary baseline survey may suggest that CRF01_AE infection was clearly related to a faster CD4+T cell loss, as compared to CRF07_BC infection. No statistically significant difference in CD4+T cell count was found between CRF01_AE and subtype B infections though the proportion of subjects with CD4+T cell <200 cells/µl in subtype B is the highest of all subtypes(χ^2^ = 0.519, *P* = 0.503).

### High Prevalence of CXCR4 Usage in CRF01_AE Infections

In this study, we used two algorithms for prediction of viral tropism analysis, algorithm I and algorithm II, in order to improve specificity as the proportion of CXCR4-tropic in CRF01_AE strains. As a whole, with these two algorithms, 26.1% (72/276) and 20.7% (57/276) of studied HIV-1 strains could be predicted to be CXCR4-tropic viruses, respectively, while all others were predicted to be CCR5-tropic viruses. Remarkably, all CRF07_BC and subtype B viruses were predicted to be CCR5-using and thus CXCR4-usage was selectively observed in CRF01_AE (χ^2^ = 55.348, *P*<0.001; χ^2^ = 52.221, *P*<0.001). Although there was little discrepancy in tropism prediction between these two algorithms, a high accordant result was observed in CRF01_AE-infected persons (40.9% vs 32.4%). No statistically significant trend in viral tropism was observed over the years (Table 2).

**Table 2:** Demographics and Clinical Characteristics Based on Co-receptor Tropism.

Patients	Algorithm I*			Algorithm II**		
	CXCR4 (%)	CCR5 (%)	*P* value	CXCR4 (%)	CCR5 (%)	*P* value
Years:						
2009 (n = 58)	10(17.2)	48(82.8)	0.069	7(12.1)	51(87.9)	0.265
2010 (n = 57)	23(40.4)	34(59.6)		16(28.1)	41(71.9)	
2011 (n = 59)	14(23.7)	45(76.3)		13(22.0)	46(78.0)	
2012 (n = 75)	18(24.0)	57(76.0)		14(18.7)	61(81.3)	
2013 (n = 27)	7(25.9)	20(74.1)		7(25.9)	20(74.1)	
Subtypes:						
CRF01_AE (n = 176)	72(40.9)	104(59.1)	<0.001	57(32.4)	119(67.6)	<0.001
CRF07_BC (n = 77)	0	77(100)		0	77(100)	
Subtype B (n = 23)	0	23(100)		0	23(100)	
CD4+T cells (cells/µl): (CRF01_AE)						
<200	16(76.2)	5(23.8)	0.006	13(61.9)	8(38.1)	0.008
200–349	16(34.0)	31(66.0)		12(25.5)	35(74.5)	
350–499	18(33.3)	36(66.7)		12(22.2)	42(77.8)	
≥500	22(40.7)	32(59.3)		20(37.0)	34(63.0)	

*Algorithm I: Geno2pheno (FPR = 10%) and webPSSM in combination;

**Algorithm II: Geno2pheno (FPR = 5%) and webPSSM in combination.

**Figure 4:**
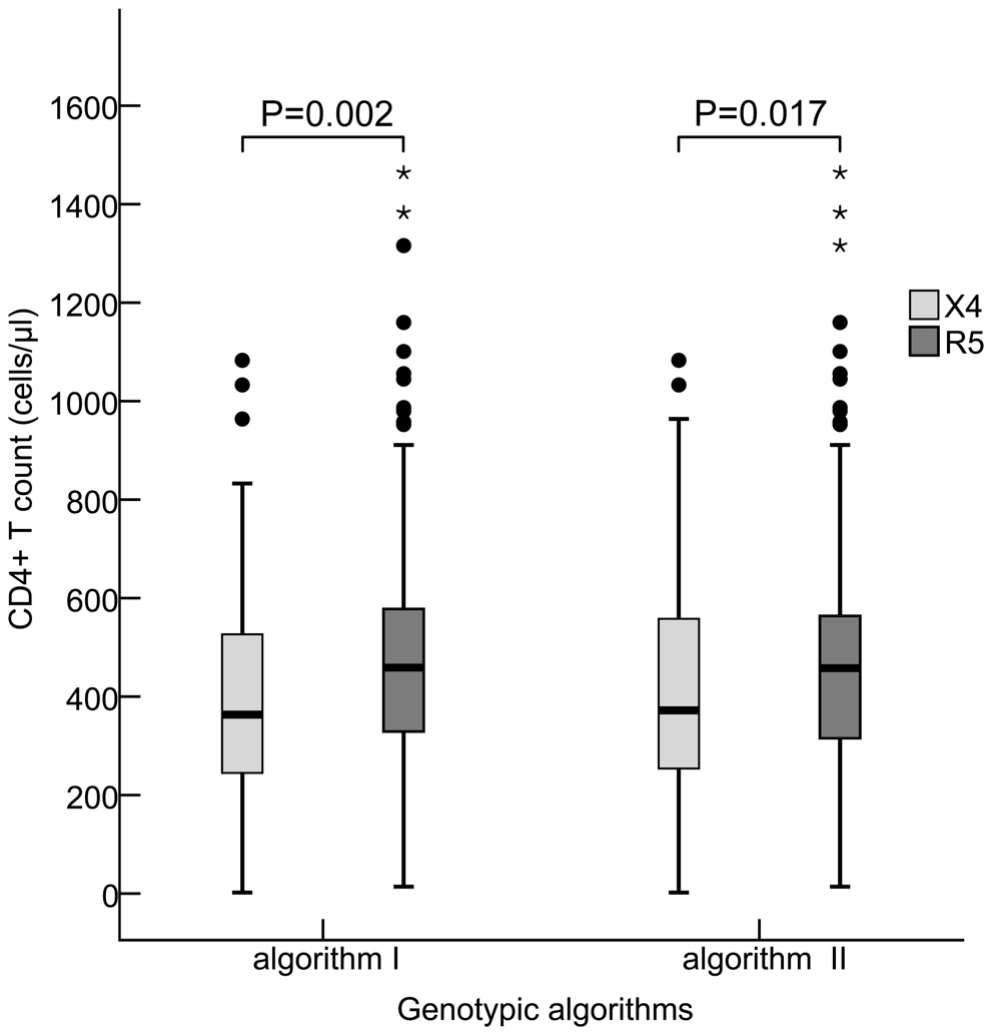
Association between HIV-1 tropisms and CD4+T cell count using two genotypic algorithms. The statistical significance of association between CXCR4- and CCR5-tropic strain infections and CD4+T cell count (Median and Interquartile Range [IQR]) was evaluated using the Two Independent Samples Nonparametric Tests (Man-Whitney U). Algorithm I: Geno2pheno (FPR = 10%) and webPSSM in combination, Algorithm II: Geno2pheno (FPR = 5%) and webPSSM in combination. X4 in light-grey square and R5 in dark-grey square were indicated as CXCR4- and CCR5-tropic strains, respectively.

Algorithm I prediction showed that persons carrying CXCR4-tropic viruses had a lower CD4+T cell count (363.5 [IQR: 243.0–533.3]) cells/µl, compared to those who were infected with CCR5-tropic virus (459.1 [IQR: 523.5–579.6]) cells/µl (Z = 3.037, *P* = 0.002). When algorithm II prediction was observed, the statistical association was similar, with a median CD4+T cell count of 361.0 (IQR: 245.0–557.5) cells/µl in the CXCR4-tropic infection compared to a median CD4+T cell count of 457.0 (IQR: 312.2–564.7) cells/µl in the CCR5-tropic infection (Z = 2.391, *P* = 0.017), (Fig. 4).

**Figure 5:**
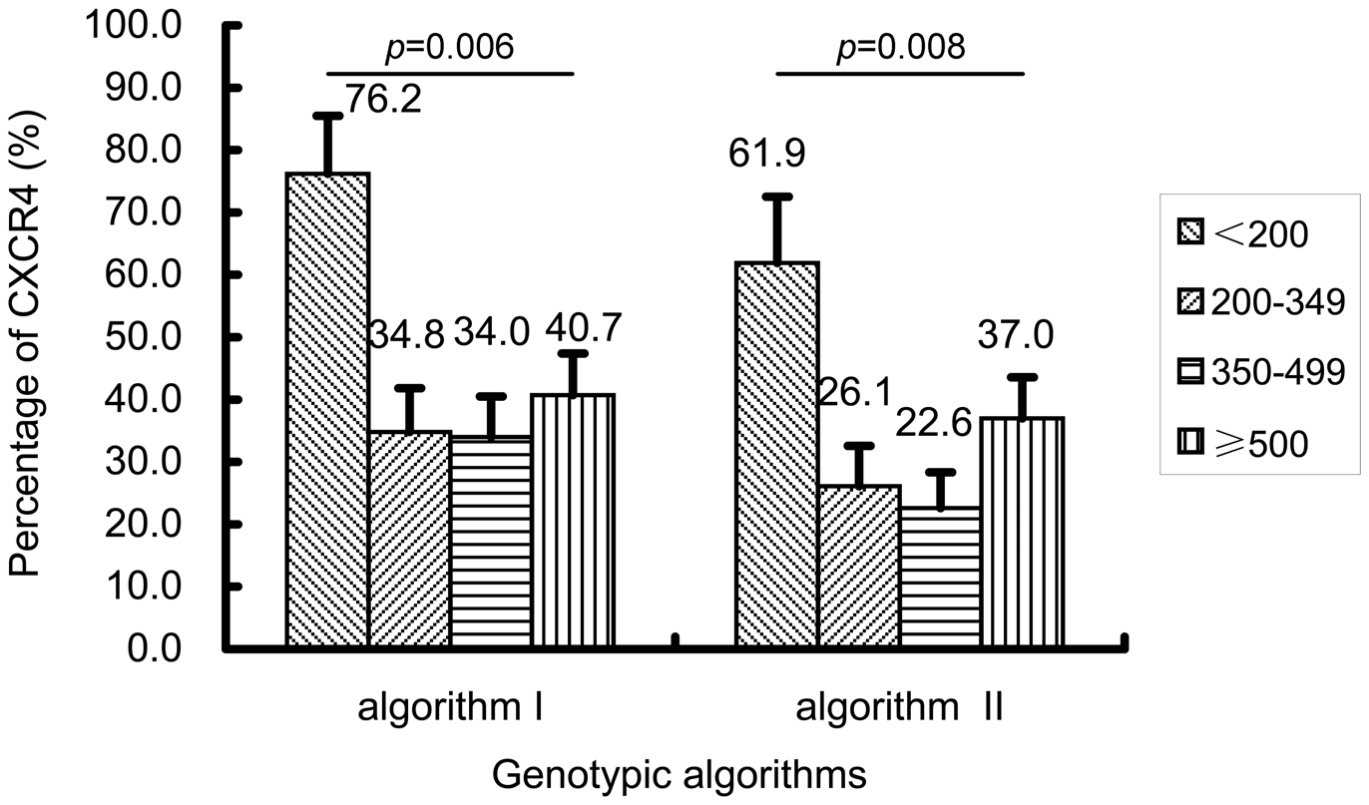
Association between CXCR4-tropic strain infection and CD4+T cell count stratified groups in HIV-1 CRF01_AE infections (n = 176) when using two genotypic algorithm predictions. The statistical significance of correlation between CXCR4-tropic strain infection and stratified CD4+T cell counts (percentage [95%CI]), when using two algorithms, was evaluated using the Fisher’s exact test.

In view of the fact that all predicted CXCR4-tropic viruses were found to be present among CRF01_AE strains (n = 176), we analyzed the relationship between CXCR4-tropic virus and different stratified CD4+T cell count. It was not unexpected that the frequencies of predicted CXCR4-tropic strain were higher in individuals with CD4+T cell count ≤200 cells/µl (76.2% in algorithm I and 61.9% in algorithm II), compared to the subjects with higher CD4+T cell counts (algorithm I: χ^2^ = 12.228, *P* = 0.006; algorithm II: χ^2^ = 11.940, *P* = 0.008). A high frequency of the predicted CXCR4-tropic viruses present in HIV-1-infected persons with a low level of CD4+T cell count ≤200 cells/µl was presumed to be partly attributed to the viral inherent pathogenic of CXCR4-tropic virus [25]. Surprisingly, high frequency of the predicted CXCR4-tropic viruses also existed in other CD4+T cell stratified groups, especially in CD4+T cell count≥500 (40.7% in algorithm I and 37% in algorithm II) (Fig 5, Table 2), similar to the observation among subtype D infection in Kenya’s study [26]. In order to exclude the potential influence derived from CD4+T cell count ≥500 on viral tropism, a supplemental analysis focusing on persons with CD4+T cell count <500 showed that persons carrying CXCR4-tropic viruses had a lower CD4+T cell counts (algorithm I: 131.0 [IQR: 77.5–162.5] cells/µl and algorithm II: 131.0 [77.5–162.5] cells/µl), compared to those who carrying CCR5-tropic virus had a higher CD4+T cell counts (algorithm I: 280.0 [IQR: 248.3–312.2] cells/µl and algorithm II: 366.0 [281.0–448.4] cells/µl) (Z = 2.851, *P* = 0.004; Z = 3.182, *P* = 0.001; respectively). However, no significant difference was found between those who were infected with CXCR4-tropic virus and CCR5-tropic virus, when CD4+T cell count ≥500.

## Discussion

This study clearly showed the presence of genetic diversity of HIV-1 circulating among young MSM population, where CRF01_AE evidently dominated. The genetic diversity of HIV-1-infected MSM would unavoidably occur due to the risky sexual behaviors [27], as can been seen in Fig. 1. The early spread of CRF01_AE was limited to the eastern coastal areas and southwest border provinces, predominantly in heterosexual populations [5], [6], [7], [8], followed by spreading to most of provinces and overtaking the subtype B [28], [29], [30], which was inferred to be the earliest circulating strain (around 1985) in the MSM population. Simultaneously, CRF07_BC infections also maintained a moderate level in different regions among MSM in China [30], [31]. In our study, 63.8% for CRF01_AE, 8.3% for subtype B and 27.9% of CRF07_BC was identified, which was consistent with previous reports [6], [29]. Additionally, all 24 second-generation recombinants related to CRF01_AE were identified that including at least 8 different recombination forms, suggesting new recombinants would inevitably occur if multi-variants were co-circulating locally. Recombination between CRF01_AE and CRR07_BC is rarely reported in China so far, but could be an alert for generation of new HIV-1 recombinant based on our finding.

Observation of differences in disease progression and transmission rate between subtypes may in part explain the changing trends in genetic subtype distribution in some populations. Surveillance of circulating subtypes and recombinants may therefore be important for epidemic prevention and control. Subtype D strain has been reported to have a higher rates of disease progression than subtype B, A, and C strains [9], likewise, recombinant BF strain was more pathogenic than subtype B strain [10]. In this retrospective investigation, low level of baseline CD4+T cell count was found among CRF01_AE strain-infected young MSM, compared to those who were infected with CRF07_BC, revealing that HIV-1 CRF01_AE could have a significant pathogenic impact on disease progression. This was supported by the previous report that the estimated annual rate of CD4+T cell loss was 58 cells/mm^3^/year (95% CI: 7 to 109, *P* = 0.027) greater in CRF01_AE infected patients compared to other infecting subtypes (mainly B) [32]. It is worth emphasizing that a high proportion of reported AIDS cases in newly diagnosed infection are not absolutely attributed to a delayed diagnosis. Our investigation evidently revealed a lowered CD4+T cell level at baseline existing among CRF01_AE among ≤25 years old recent infections, in which baseline CD4+T cell count≤200 cells/µl was found up to 12.1%, strongly suggesting that the pathogenicity of CRF01_AE strain might play a significant role in disease progression. Two recent studies from northern Thailand with cohorts of CRF01_AE infected patients also reported a 3-year shorter median survival compared to age-matched individuals for the Western European CASCADE cohort, where subtype B predominates [33], [34]. Furthermore, based on the Chinese officially reported data between 2011 and 2012, the proportions of AIDS cases in newly diagnosed infections in Guangxi (where CRF01_AE predominates) and Xinjiang (where CRF07_BC predominates) were 37.2% and 14.1%, respectively, and the cumulative mortalities were 24.7% and 15.5%, respectively, in corresponding areas, which provided an indirect evidence that CRF01_AE-infected individuals might have the trait for a faster disease progression. Remarkably, we did not find the significant difference in baseline CD4+T cell count between CRF01_AE and subtype B infections, however strong evidence would be still required with a larger cohort of patients infected with these two subtypes. The subtype- and recombinant-related biological mechanisms involved in this phenomenon remain unknown and could be essentially virological aspects, such as co-receptor tropism, transmissibility, or replicative capacity, or could be related to host immunity, such as bearing gp120 molecules more able to bind CD4 or less exposed to antibodies, or carrying immunodominant CTL epitopes that fail to elicit efficient CD8+T cell-mediated response [10].

Overall, the prevalence of CXCR4 viral tropism in this studied population was 26.1% (algorism I) and 20.7% (algorism II). As mentioned above, viral co-receptor tropism could be one of pathogenic mechanisms among different HIV-1 subtypes or recombinants. In order to reduce the overestimation of the presence of CXCR4-tropic virus [23], we used two tropism prediction algorithms to evaluate the prevalence of CXCR4 tropism in CRF01_AE strains circulating among MSM of ≤25 years old and showed 40.9% (Algorithm I) and 32.4% (Algorithm II), respectively, which was similar to Belgium and Singapore’s studies [11], [12]. Although the prevalence of CXCR4-tropic virus in subtype B samples is around 10–20% in many validated global studies [11], [12], [23], unexpectedly, none of CXCR4-tropic virus was observed in subtype B with a higher proportion of CD4 T cell<200 in our locality, possibly due to smaller numbers of subjects studied. A large cohort study of viral tropism in relation to subtype B needs to be conducted in China. Subjects with CXCR4-tropic virus had a lower CD4+T cell count compared to CCR5-tropic virus has been demonstrated by several studies [11], [12]. The high frequencies of CXCR4-tropic strain in the level of CD4+T cell count ≤200 cells/µl limited to CRF01_AE-infected individuals (algorism I: 76.2% and algorism II: 61.9%), compared to other stratified CD4+T cell counts, maybe partly attributed to the inherent pathogenicity of CXCR4-tropic virus. This also was confirmed by the phenomenon that the higher prevalence of CXCR4-usage among CRF01_AE-infected subjects may experience faster CD4+T cell loss compared to subjects infected with CRF07_BC (Fig 2). The high level presence of CXCR4-tropic virus among recent infections with CRF01_AE could have clinical implications and therefore early co-receptor tropism screening and early treatment of those who carry CXCR4-tropic CRF01_AE strain are necessary in order to prevent fast immune deterioration and halt the transmission of these strains. It is also well known that the emergence of CXCR4-tropic variants tends to occur much later as disease progresses [35], and however to our surprise, a high frequency of the predicted CXCR4-tropic virus was also observed in some infected persons with CD4+T cell count ≥500 cells/µl (40.7% in algorithm I and 37% in algorithm II) among CRF01_AE-infected subjects. Nevertheless, no significant difference in baseline median CD4+T cells was found between those who were infected with CXCR4-tropic virus and CCR5-tropic virus when CD4+T cell count ≥500. Conversely, there were significant differences between those who infected CXCR4- and CCR5- tropic viruses when their CD4+T cell count ≤500 (algorism I: *P* = 0.004 and algorism II: *P* = 0.001, respectively). We presume that these persons infected by CXCR4-tropic virus still stayed a period of the relatively stable and higher CD4+T cell counts after a acute infection because of the different immune status among the MSM individuals.

Large epidemiologic cohort studies demonstrated that early infection probably accounts for up to two-thirds of transmission events and the overall prevalence of X4/DM viruses in early infection fluctuates between 10% and 20% [11], which is supported by our findings. Although numerous studies have attempted to correlate the predominance of CCR5-tropic strains during the early stage of infection with a biological bottleneck inherent to the genital mucosa [36], no conclusive evidence has been provided to indicate that CXCR4-tropic viruses were less transmissible. Chalmet et al. recently reported that 11% of 63 transmission clusters identified in 539 newly diagnosed infections resulted from CXCR4-tropic viruses transmission [11], implying that like CCR5, CXCR4 virus could be transmitted as a result of a stochastic process. Our finding that a high frequency of CXCR4-tropic virus in some persons with CD4+T cell count ≥500 could be partly as a result of recently direct transmission of CXCR4-tropic virus among MSM, as being a vulnerable and most-at-risk population with a relatively brittle gut mucosal barrier. Recent finding suggested that CCR5- and CXCR4-tropic subtype C HIV-1 isolates might have equal transmission fitness but reduced pathogenic fitness relative to other group M HIV-1 isolates [37]. Therefore, these two dominating strains in China, CRF01_AE and CRF07_BC, would be of difference in clinical outcome in HIV-1-infected. Information on reliability of Geno2pheno and/or webPSSM for the prediction of co-receptor use in CRF01_AE remains sparse, we still can not rule out the possibility that our conclusions result in part from an overestimation in the prediction of CXCR4 use in CRF01_AE. Despite this possible bias, our finding do have important consequences and warrant further investigation. We here did not use phenotypic assay to compare with *env* V3-based Geno2pheno and webPSSM in combination, because of a quite laborious, expensive, and time-consuming. Moreover, a recent study from Hong Kong suggested that a better genotypic tropism prediction for HIV-1 CRF01_AE would be using Geno2pheno and webPSSM algorithms in combination with 88.9% and 89.3%, respectively, for sensitivity and specificity, when phenotypic data was compared [24].

This study focused on young MSM under 25 years old, with an average age 22.7 years old. However, we would not exclude the possibility of the presence of new or recent infections in MSM over 25 years old. Extensive and intensive investigations of HIV-1 subtypes- and recombinants-related pathogenic survey in different ages remain vital and necessary in China for implementation of a novel antiretroviral therapy strategy and efficient transmission intervention.

In conclusion, this study for the first time in China revealed that CRF01_AE strain with a high frequency of CXCR4-tropism has been circulating in young MSM population, which might cause a severe loss of CD4+ T cell count and speed up disease progression, compared to CRF07_BC strain. A regular surveillance of HIV-1 genetic subtypes, CD4+ T cell count and viral co-receptor usage would be greatly beneficial for effectively monitoring disease progression, improvement of antiretroviral strategy and prompt intervention of transmission.
