# Correction: Evidence that HIV-1 CRF01_AE is Associated with Low CD4+T Cell Count and CXCR4 Co-receptor Usage in Recently Infected Young Men Who Have Sex with Men (MSM) in Shanghai, China

**DOI:** 10.1371/journal.pone.0097395

**Published:** 2014-05-06

**Authors:** The PLOS ONE Staff

There were errors in the Funding section. The correct funding statement is as follows: “This work was supported by the National Science and Technology Major Project for Infectious Disease Control and Prevention (2012-ZX10001-002) and National Natural Science Foundation of China (81373060). The funders had no role in study design, data collection and analysis, decision to publish, or preparation of the manuscript.”

The fourth column header in the first line of Table 1 is incorrect. The correct header is: Subtype B (%).
